# Where Do the Rural Poor Deliver When High Coverage of Health Facility Delivery Is Achieved? Findings from a Community and Hospital Survey in Tanzania

**DOI:** 10.1371/journal.pone.0113995

**Published:** 2014-12-02

**Authors:** Manuela Straneo, Piera Fogliati, Gaetano Azzimonti, Sabina Mangi, Firma Kisika

**Affiliations:** 1 Doctors with Africa, CUAMM, Iringa, Tanzania; 2 Tosamaganga Council Designated Hospital, Iringa, Tanzania; 3 Reproductive and Child Health, Council Medical Office, Iringa District Council, Tanzania; Örebro University, Sweden

## Abstract

**Introduction:**

As part of maternal mortality reducing strategies, coverage of delivery care among sub-Saharan African rural poor will improve, with a range of facilities providing services. Whether high coverage will benefit all socio-economic groups is unknown. Iringa rural District, Southern Tanzania, with high facility delivery coverage, offers a paradigm to address this question. Delivery services are available in first-line facilities (dispensaries, health centres) and one hospital. We assessed whether all socio-economic groups access the only comprehensive emergency obstetric care facility equally, and surveyed existing delivery services.

**Methods:**

District population characteristics were obtained from a household *community survey* (*n = 463*). A *Hospital survey* collected data on women who delivered in this facility (*n = 1072*). Principal component analysis on household assets was used to assess socio-economic status. Hospital population socio-demographic characteristics were compared to District population using multivariable logistic regression. Deliveries' distribution in District facilities and staffing were analysed using routine data.

**Results:**

Women from the hospital compared to the District population were more likely to be wealthier. Adjusted odds ratio of hospital delivery increased progressively across socio-economic groups, from 1.73 for the poorer (p = 0.0031) to 4.53 (p<0.0001) for the richest. Remarkable dispersion of deliveries and poor staffing were found. In 2012, 5505/7645 (72%) institutional deliveries took place in 68 first-line facilities, the remaining in the hospital. 56/68 (67.6%) first-line facilities reported ≤100 deliveries/year, attending 33% of deliveries. Insufficient numbers of skilled birth attendants were found in 42.9% of facilities.

**Discussion:**

Poorer women remain disadvantaged in high coverage, as they access lower level facilities and are under-represented where life-saving transfusions and caesarean sections are available. Tackling the challenges posed by low caseloads and staffing on first-line rural care requires confronting a dilemma between coverage and quality. Reducing number of delivery sites is recommended to improve quality and equity of care.

## Introduction

The majority of maternal deaths are concentrated in limited resources countries, and within them the poorest bear the greatest burden [1]. This inequity has been linked to reduced access of the rural poor to professional delivery services. Underlying factors are limited health services' availability or accessibility in rural areas and lower demand by the population [2], [3].

Timely access to skilled attendance at birth is essential to maternal mortality reduction [4]. In sub-Saharan Africa, in practise, this is only available in health facilities [5]. The type of obstetric care offered varies, ranging from the full comprehensive emergency obstetric care (c-EmOC) package, which includes caesarean sections and blood transfusions [6], generally only available in hospitals, to lower, variable levels of care in first-line facilities.

To reduce mortality among the rural poor, the focus of the international public health community has been on improving coverage of institutional delivery [7], though the evidence of the effectiveness of this intervention is scarce [8]. As Millennium Development Goal 5-related strategies take effect, coverage of institutional deliveries will inevitably improve. Will all socio-economic groups equally benefit from high coverage? Limited available data appear to indicate that this may not be so. In Indonesia for example, primary health care has been strengthened by ensuring every village has a midwife. The strategy was successful in ensuring access of all socio-economic groups to skilled attendance at birth, however poorer women remained disadvantaged in accessing caesarean sections [9]. Trends of caesarean sections across different countries have shown that the poorest consistently lower access to this potentially life-saving procedure [10]. In Tanzania, Ferry et al [11] found inequity in access to inpatient care at health centre level, in spite of existing fee exemptions for children, pregnant women and the elderly.

This issue is central to planning of maternal services in limited resources countries beyond the basic objective of coverage. Lack of quality and equity of services represent barriers to achieving maternal mortality reduction, particularly among the poorest [12].

The question was addressed in Iringa District, a rural District in Tanzania, where high coverage of institutional delivery has been achieved. Data from this paradigmatic area can help to understand ahead of time what will happen in limited resources countries as coverage improves. The United Republic of Tanzania is no exception to the high maternal mortality ratios in sub-Saharan Africa in spite of a well developed primary health care network. A ratio of 417.5 deaths per 100 000 live births is estimated corresponding to 7990 deaths countrywide [13]. The health system is organized at District level and includes dispensaries, health centres and hospitals [14]. Women can obtain assistance at childbirth in all types of facilities. The former two are first-line facilities where delivery care is part of reproductive and child services, though staffing is often insufficient [15]. Delivery services are free of charge according to Tanzanian government policy [11], [16]. Though nationwide home deliveries remains common (52%) [17], a district-representative household survey carried out in 2009 in Iringa District (unpublished) documented 88% of facility births among women with a recent delivery. These data were confirmed by the 2010 Tanzanian Demographic and Health Survey findings (institutional deliveries for the whole Iringa region 80.4%) [17]. High coverage in the area is linked to a high facility density (3629 people per first-line facility), nearly double that of the country as a whole (6064 per facility) [14].

The overall aim of the study was to investigate how different socio-economic classes access delivery services in an area of high coverage of institutional deliveries in order to draw conclusions on childbirth services for the rural poor. To complete the picture, we assessed existing obstetric services in the District, in particular with regards to distribution of deliveries between first-line and secondary care facilities, as these factors are crucial for the overall objective of improving care and reducing mortality. The results of the study will provide insights to address maternal health policies in limited resources countries.

## Methods

### Study setting

Iringa District (formerly Iringa rural) is within Iringa Region, in the Southern Highlands of Tanzania. The population according to the 2012 census was 254,023 [18]. It is mostly rural, with 85% relying on subsistence farming. The District includes 122 villages. Health services in 2012 were available in 73 facilities, of which 66 dispensaries, 6 health centres and one diocesan District hospital [19]. The majority of health facilities are public, with only 27% run by private non-profit organizations.

### Baseline population data

Baseline information on the District population including socio-economic data was obtained from a cross-sectional household survey carried out in October 2009 (*Community survey*). Objective of the survey was to collect information on access to health services in the area, as part of a health system strengthening programme. A representative sample of the District population was obtained through two-stage cluster sampling. Thirty villages were randomly chosen in the first stage with probability proportional to size. Twenty-five households were selected in each village in the second stage through random systematic sampling. Women with a delivery in the last five years were included in analysis.

### Data on women who accessed hospital delivery services

The socio-economic profile of women accessing the only comprehensive emergency obstetric care facility was obtained from a cross-sectional survey of women discharged from the District hospital Maternity Ward between October 2011 to May 2012 (*hospital survey*). The survey was conducted as part of a development programme, on access, quality and equity of maternal services. Interviews at discharge were conducted in Swahili by female trained interviewers using a pretested structured questionnaire. For women who had died, information was collected from relatives. In the hospital survey, only women who had delivered in the hospital and lived within the District were included in analysis. Women from outside the District (who have travelled beyond their District hospital) may belong to a higher socio-economic group, therefore creating bias in the analysis.

As validation of collected data, records collected were matched with hospital Maternity registers.

### Data entry and analysis

Data entry and cleaning was carried out using Epidata software (version 3.1) by a principal investigator, and analysis was carried out using STATA (version 9) software.

### Baseline characteristics of the two populations

Characteristics of women from the two surveys were compared (Dataset S1).

Variables examined were age (at index delivery for the community survey), parity, education, sex of household head, type of delivery and socio-economic status. Proportions and 95% CI were estimated for both populations taking study design into account. After merging of data sets, bivariate and multivariable analysis were performed. Crude odds ratios for belonging to the hospital population were produced, with a 95% confidence interval.

Multivariable logistic regression including all variables significant in bivariate analysis was performed to estimate adjusted odds ratios of belonging to the hospital compared to the District population. Svyset commands were used to account for clustered design.

### Socio-economic stratification

Socio-economic status (SES) was assessed based on durable household possessions (bicycle, radio, mobile phone) and housing characteristics (non-grass roof, non-mud floor and electricity) as applied by Bernard et al [20] in rural Tanzania. Principal Component Analysis was used to define weights to each variable and to construct a household socio-economic score for the community and for the hospital population respectively [21]. Although the score from the first principal component does not give information on absolute level of wealth, it can be used for comparison across different settings, provided that calculation is based on the same variables [21].

We classified the district population into five SES groups (1–5), from poorest to richest by dividing the community household socio-economic score into quintiles. We thus applied the quintile cut-off values derived from the community sample to the hospital population socio-economic score to create five comparable SES categories across the two settings.

### Availability and utilization of District obstetric services

Details of available services and the distribution of deliveries within them in 2012 were obtained using the routine District Health Management Information System (HMIS, MTUHA in Tanzania), through the District Medical Office.

Annual reports are compiled by each health facility on a national standardized form (F005), and sent to the District Medical Office yearly. The annual reports for 2012 for the facilities of Iringa District were examined, and data on deliveries was collected (Dataset S2). Reported data was cross checked with data at facility level during supervision visits.

### Human resources availability

Data on health facility staffing in 2012 was obtained from the Human Resources Information System, available in the District Medical Office (Dataset S2). The information was validated and where necessary updated during health facility supervision visits.

Skilled birth attendants (SBA) are accredited health professionals with the necessary skills to manage childbirth and to identify, manage and refer complications in women and newborn [22], [23]. In Tanzania, clinicians (medical officers, assistant medical officers, clinical officers), and enrolled and registered nurses are classified as skilled birth attendants; lower cadres such as nursing assistants are not [24].

### Ethical statement

Ethical clearance for the study was obtained from the National Institute for Medical Research, Dar es Salaam, Tanzania. Participants to both community and hospital surveys provided signed informed consent.

## Results

**Figure 1:**
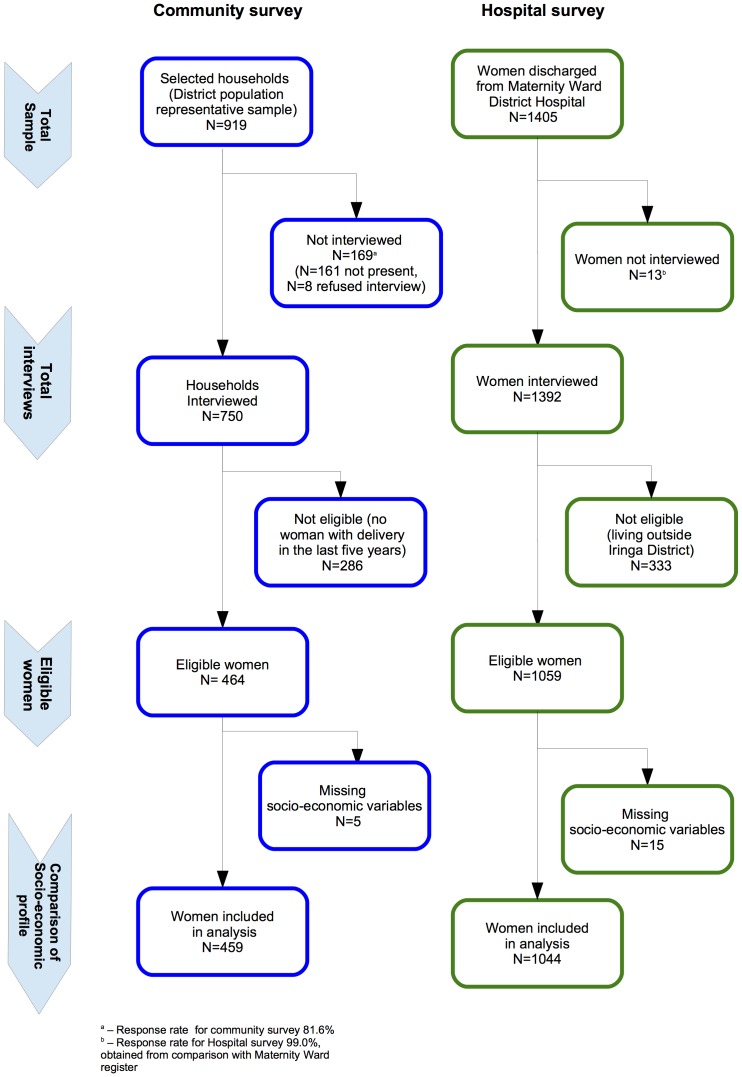
Data flow for the community and hospital surveys.

The flow chart in Figure 1 summarizes data collection.

### District population data

In the community survey, 919 households were visited: inhabitants were absent in 161, and in 8 refused the interview (response rate 81.6%). In the interviewed households, there were 463 women with a recent delivery, and their data was analysed for this study. Mean age at delivery was 28.0 years (95% CI, 27.3–28.6), and median parity was 3 (2–5). Most belonged to the Hehe or Bena tribes (81.1%, 95% CI, 74.6–87.7), only 0.2% (95% CI, 0–0.6%) were semi-nomadic Masai, and the rest belonged to other tribes (18.7%, 95% CI, 12.2–25.1).

### Hospital survey

1072 women living within the District were interviewed. In the study period, by comparison to the Maternity Ward register, 99.0% of women admitted were interviewed. Women living outside the District were excluded from analysis. Mean age was 25.7 years (95% CI, 25.4–26.1) and median parity was 2 (range 1–4). Here too, most women were Hehe/Bena, 86.1% (95% CI, 84.0–88.2); Masai were 0.9% (0.4–1.5), the rest belonged to other tribes (12.9%) (95% CI, 10.9–15.0).

Out of 1072 childbirths, 34 were twin deliveries, with a total of 1106 newborn. 1071 were born alive, the remaining were either fresh (16) or macerated stillborn (19). 1034 newborn were discharged alive while 37 died (36 within 7 days, 1 after the first week). Early neonatal mortality was 33.6 per 1000 live births (95% CI, 30.2–37.3).

During the survey period, 6 women died, of which 5 due to direct obstetric causes (Eclampsia 3, infection 1, septic shock 1).

1043 women provided information on the reason for hospital delivery. The most frequent answer was “I chose myself” to deliver in the higher level facility (55.0%, 95% CI, 52.0–58.1). 37.7% (95% CI, 34.7–40.6) were advised on hospital delivery during antenatal care, only 5.6% (95% CI, 4.2–7.0) were referred during labour. The remaining were either advised by relatives/husband, or had attended ANC in the hospital.

### Comparison of the two populations

Table 1 compares baseline characteristics of the two populations. There were differences in frequency distribution of age, parity, education, sex of household head, type of delivery and socio-economic status.

**Table 1:** Socio-demographic characteristics of women who delivered at District hospital compared to women from the community of provenance.

Variable	Community survey (N = 463)			Hospital survey (N = 1072)		
	*n*	%	(95% CI)*	*n*	%	(95% CI)
**Age (years)**						
≤19	*38*	8.3	(5.4–11.3)	*176*	16.4	(14.2–18.7)
20–39	*395*	86.6	(83.2–90.1)	*868*	81.1	(78.8–83.5)
≥40	*23*	5.0	(2.8–7.3)	*26*	2.4	(1.5–3.4)
**Parity**						
1	*74*	16.1	(12.0–20.1)	*429*	40.1	(37.1–43.0)
2–4	*257*	55.7	(50.7–60.8)	*445*	41.5	(38.6–44.5)
≥5	*130*	28.2	(22.6–33.8)	*197*	18.4	(16.1–20.7)
**Education (years)**						
0	*58*	12.5	(9.0–16.1)	*74*	7.0	(5.5–8.6)
1–6	*35*	7.6	(4.8–10.3)	*22*	2.1	(1.2–3.0)
7	*353*	76.2	(71.5–81.0)	*821*	78.0	(75.5–80.5)
≥8	*17*	3.7	(1.2–6.2)	*135*	12.8	(10.8–14.9)
**Sex of household head**						
Male	*412*	89.4	(86.0–92.8)	*965*	92.6	(91.0–94.2)
Female	*49*	10.6	(7.2–14.0)	*77*	7.4	(5.8–9.0)
**Type of delivery**						
Vaginal	*420*	90.7	(87.3–94.1)	*736*	68.7	(65.9–71.5)
Cesarean section	*43*	9.3	(5.9–12.7)	*335*	31.3	(28.5–34.1)
**SES**						
Very low	*91*	19.8	(14.2–25.5)	*64*	6.1	(4.7–7.6)
Low	*92*	20.0	(15.6–24.5)	*120*	11.5	(9.6–13.4)
Medium	*84*	18.3	(14.8–21.8)	*180*	17.2	(14.9–19.5)
High	*88*	19.2	(14.5–23.8)	*252*	24.1	(21.5–26.7)
Very high	*104*	22.7	(17.3–28.0)	*428*	41.0	(38.0–44.0)

*adjusted for cluster design.

Iringa District, Tanzania. 2009–2012.

Crude and adjusted odds ratios, with 95% confidence intervals and p values, of belonging to the hospital population compared to the District population are shown in Table 2.

**Table 2:** Association between covariates. Study population from hospital survey compared to the study population from community survey.

Variable	OR crude	(95% CI)	p-value*	OR adjusted	(95% CI)	p-value*
**Age (years)**						
≤19	2.11	(1.44–3.09)	0.0004	1.15	(0.74–1.79)	0.5159
20–39	1	-	-	1	-	-
≥40	0.51	(0.32–0.82)	0.0061	0.63	(0.37–1.06)	0.0798
**Parity**						
1	3.35	(2.51–4.47)	0.0000	3.45	(2.58–4.61)	0.0000
2–4	1	-	-	1	-	-
≥5	0.88	(0.67–1.15)	0.3204	1.23	(0.91–1.66)	0.1810
**Education (years)**						
0	0.55	(0.39–0.76)	0.0008	0.89	(0.62–1.28)	0.5187
1–6	0.27	(0.18–0.40)	0.0000	0.35	(0.23–0.54)	0.0000
7	1	-	-	1	-	-
≥8	3.41	(1.71–6.81)	0.0010	1.43	(0.72–2.87)	0.2973
**Sex of household head**						
Female	1	-	-	1	-	-
Male	1.49	(1.04–2.13)	0.0306	2.10	(1.16–3.81)	0.0163
**Type of delivery**						
Vaginal	1	-	-	1	-	-
Cesarean section	4.45	(2.98–6.63)	0.0000	4.23	(2.88–6.21)	0.0000
**SES**						
Very low	1	-	-	1	-	-
Low	1.85	(1.34–2.56)	0.0005	1.73	(1.22–2.45)	0.0031
Medium	3.05	(2.00–4.64)	0.0000	2.58	(1.63–4.09)	0.0002
High	4.07	(2.57–6.45)	0.0000	3.54	(2.17–5.78)	0.0000
Very high	5.85	(3.78–9.06)	0.0000	4.53	(2.86–7.17)	0.0000

*Adjusted Wald test.

The youngest age group appeared more likely in bivariate analysis to be part of the hospital population (OR 2.11, p<0.001). The oldest age group [≥40) appeared less likely to be part of the hospital population (OR 0.51, p = 0.0061). These differences were however not significant in multivariable analysis.

Women with a first pregnancy were more represented in the hospital, with the difference remaining significant in multivariable analysis (adjusted OR 3.45, p<0.001). Unexpectedly, women of high parity (≥5) were not more represented in the hospital after adjusting for other variables (OR 1.23, p>0.05).

OR for education levels were inconsistent. Only women with incomplete primary education (1–6 years) were significantly less represented in the hospital (OR 0.35, p<0.001), compared to women with complete primary education (7 years). For women with no formal education, adjusted OR was not significant. When education was dichotomized (complete primary or greater versus none/incomplete primary ≤6 years), the adjusted odds ratios was 2.66 (95% CI 1.09–1.99, p = 0.0125) (data not shown).

When the households were male-headed, the OR was double compared to female-headed ones (adjusted OR 2.10, p = 0.0163).

As expected, the odds of Caesarean section was significantly greater for the hospital population (OR 4.23, p<0.001).

**Figure 2:**
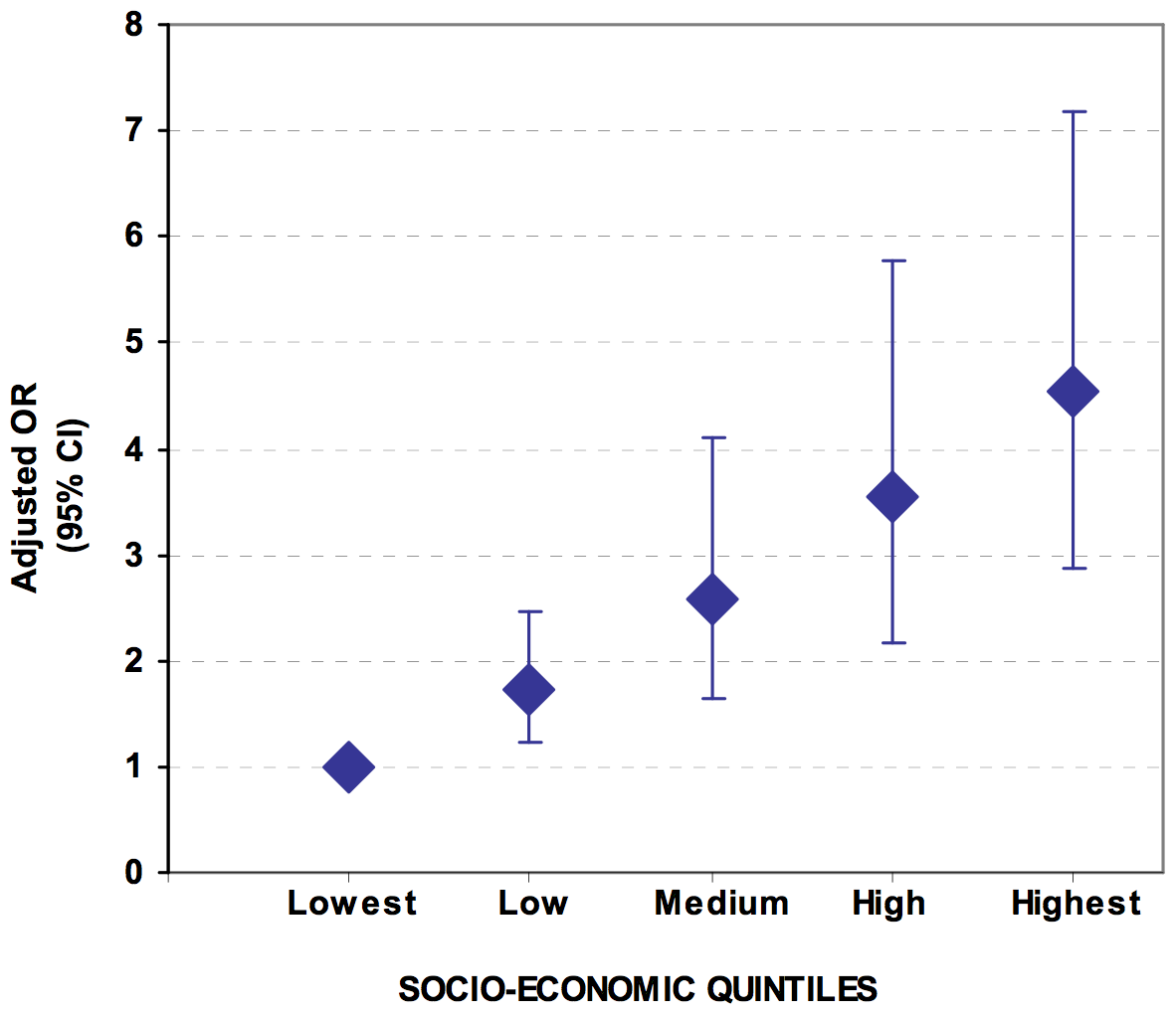
Odds ratios of belonging to hospital population compared to District population by socio-economic quintiles, with respective 95% confidence intervals. Iringa District, Tanzania. 2009–2012.

In adjusted analysis, OR increased progressively across SES categories, compared to the baseline of the poorest group (OR 1.73 up to 4.53), indicating women are increasingly more represented in the hospital as household poverty decreases (Figure 2).

### District obstetric services

7645 institutional deliveries were recorded in 2012 in Iringa District Health Management Information System. 5505 (72%) took place in 68 first-line facilities, and 2140 (28%) in the District Designated hospital. 2 dispensaries did not provide reproductive services, and no data was available for two dispensaries.

In health centres, deliveries ranged from 56–277, with a median of 158.5. In dispensaries, the number ranged from 2–203, with a median of 62.5.

The distribution of deliveries by facility caseload in Iringa District has been summarized in Table 3.

**Table 3:** Distribution of deliveries by facility caseload in Iringa District in 2012 (based on HMIS data).

Facility delivery caseload/year	Deliveries (%)	N. facilities (%)
0–50	684 (8.9)	21 (30.9)
51–100	1840 (24.1)	25 (36.8)
101–150	1423 (18.6)	12 (17.6)
151–200	854 (11.2)	5 (7.4)
201–250	427 (5.6)	3 (4.4)
251–300	277 (3.6)	1 (1.5)
>300	2140 (28.0)*	1 (1.5)*
**Total**	**7645**	**68**

*District Hospital.

Overall, approximately one third (2524/7645, 33.0%) of deliveries took place in facilities with caseload ≤100/year.

### Human resources

68 first-line facilities had a total of 191 health workers in 2012, of which 140 were skilled birth attendants. Health workers in the hospital were 139, of which 81 were SBA. Numbers in the hospital were calculated on the whole institution, not simply on the Maternity Ward.

In health centres, health workers ranged from 5–13, median 10.5; skilled birth attendants' range was 3–9, median 6.5. In dispensaries, total health workers were 1–7, with a median of 2; available SBA were 0–5, with a median of 1.5. There were less than two SBA in 28/68 (40%) first-line facilities, all of which were dispensaries. Thus, nearly half of the dispensaries (28/62 dispensaries, 45%) had insufficient staff to provide maternal services 24 hourly.

## Discussion

Evidence from this study indicates that poorest women are accessing lower level health services for delivery, which offer worse quality of care, due to limited caseloads and poor staffing. Two major findings support this. The first is that the poorest women are disproportionately under-represented in the hospital, the only facility able to provide all functions of comprehensive emergency obstetric care. There is a gradient across socio-economic groups, with increasing odds of hospital delivery as wealth increases. The second finding emerges from analysis of available services: deliveries are remarkably dispersed over poorly staffed facilities.

The findings will be discussed separately.

### Access of socio-economic groups to c-EmOC facility

Poorer women are disadvantaged in access to higher level delivery services. There are two implications to this finding. The first is that women from the lower SES groups are less likely to deliver where blood transfusions and caesarean sections are available. They are thus disadvantaged with regards to potentially life-saving procedures. The second implication is that these women, in this context, are more likely to receive childbirth care in first-line facilities.

An additional finding which has emerged is that women mostly access comprehensive emergency obstetric care by choice, bypassing the first-line facility. This may appear in contrast to the findings by Kruk et al [7]. In their study on women's bypassing of the nearest facility in Western Tanzania, no association with wealth was observed. The latter study was carried out in a low facility coverage context (<40% of facility deliveries), thus very different from the described in this manuscript, and examined by-passers to any health facility (including same level facilities). Only half the by-passers (93/186) had accessed a hospital [7]. Our study, focused on by-passers to the c-EmOC facility, and suggests poorer women bypass less. Greater education, economic resources, and smaller family sizes probably contribute to wealthier women's preference for delivery in a higher level facility.

### Distribution of deliveries and human resources for health

Additional important information to understand where the poorest deliver is contributed by analysis of District delivery services. As noted previously, excellent coverage is associated to high facility density. Just over a quarter of institutional deliveries had taken place in the c-EmOC facility, with the rest in first-line facilities. One third of childbirths were attended in facilities with very low delivery volume (<100 deliveries per year). In the framework of the Primary Health Care Development Programme, the Tanzanian government aims to expand health facilities, with a dispensary in every village and a health centre in every ward (an administrative area with approximately 50000 people) [25], which corresponds to approximately doubling the dispensaries (n = 5607 in 2011), and quadrupling the health centres (n = 684 in 2011) [14]. Development of primary care in Tanzania has been successful in shortening the distance between users and services. For example, living within 5 km of a dispensary has a documented advantage on under-five survival [26]. The consequence of the expansion of primary health care on childbirth care is that deliveries are dispersed over a large number of facilities. There is not much available evidence on the optimal number of deliveries to be assisted per year to ensure quality, though a caseload greater than 150 per year has been suggested [24].

Severe shortage of qualified staff in the District completes the picture. Human resources for health shortage in resource limited countries has been repeatedly reported, and is considered a major obstacle to achieving MDGs [14]. The gap for all cadres in Tanzania was estimated at 64% in 2012/13 [14]. Though it has reduced from 72% in 2006, it is unlikely to be filled in the short term, considering the planned expansion of facilities. Furthermore, absences from work stations are frequent, due to annual leave, sickness, travel to District offices or trainings [27], [28]. One survey in Tanzania found 49% of nurses absent from the work station [27]. As part of the “workload indicator of staffing needs” WHO tool [29], an available working time (“the time a health worker has available to do his/her work, taking into account authorized/unauthorized leave”) of 202 days per year was reported in an urban context in Tanzania [28]. In rural areas, absences are likely to be more frequent, due to longer travelling times, thus reducing available working time further. On the basis of days present at the work station only, to ensure full-time delivery care there is a requirement of at least two skilled birth attendants. 40% of first-line facilities (45% of dispensaries) did not have sufficient skilled birth attendants to ensure full-time delivery care.

### Maternal health policy implications

The detailed picture provided by this study in a limited geographical area highlights a conflict between coverage and quality of delivery care in rural contexts. The study's findings can provide useful insights to adjust maternal health policy without compromising accessibility, and can be used to extrapolate recommendations applicable to other limited resources contexts.

We argue that delivery is inherently different from other primary health care activities, as it cannot be planned and may be associated to life-threatening complications. For effective coverage, care must be offered every day, 24-hourly, which is hindered by the shortage of qualified staff. Complications must be handled by staff with the necessary knowledge, skills and equipment. Though gaps in equipment and knowledge can be filled even at peripheral level, low caseloads hinder staff from maintaining the necessary skills.

An adjustment of policy on where childbirth care is available is a possible solution. A reduction in the number of delivery sites offers several advantages. 1. Greater quality of care. A limited number of first-line facilities could be upgraded to delivery sites, with adequate staffing to provide full time care, and a planned caseload greater than 150/year, to ensure staff maintain knowledge and skills through practise. Other first-line facilities would continue to provide other maternal care services, such as antenatal and postnatal care, but not delivery care. 2. More rational and efficient distribution of scarce human resources for health, as the latter facilities would require fewer human resources to function. 3. A likely improvement in cost-effectiveness of delivery care in limited resources countries. There are two potential disadvantages of the proposed policy change. The first is a reduction of women's access to delivery services, due to greater traveling distances; the second, local opposition, due a reduction of services available.

Further studies in Iringa District on health facilities' accessibility using a geographical information system could provide valuable, more detailed information on women's utilization of services. This mapping could contribute, together with other local factors, to identifying potential delivery sites among existing facilities. A small scale study in a limited geographical area on the outcome of reduced numbers of delivery sites could provide useful information to policy makers.

### Strengths and limitations of the study

To the authors' knowledge, this is the first demonstration of inequity of access to the full package of obstetric services where excellent institutional delivery coverage has been achieved. The study's greatest strength stems from the comparison of the socio-economic profile of women in the c-EmOC facility with women from the population of origin, not more general regional data from a Demographic and Health survey.

In addition, data was collected from first-line health facilities and a secondary facility in the same District, allowing more detailed analysis than when lower level facility data is compared with data from the country as a whole. The study's high response rate is an additional strong point.

There are limitations to the study which should be considered. The first is that the two populations analysed are not time-matched. The surveys were part of two different studies, and community data was collected in 2009, while in the hospital 24 to 31 months later. The main criticism could be that availability of household assets (such as mobile phones) may have varied in the country. Though mobile phone use has increased in Tanzania, power availability in rural areas may not have increased in the same way.

A second possible criticism is on the validation of data collected for socio-economic stratification. Though both surveys used respondents' answers to a questionnaire, in the first survey interviewers visited women's homes, therefore were able to confirm directly availability of some assets (such as floor and roofing materials); in the second survey, no direct observation was possible, as interviews were conducted in the hospital. Though some degree of social desirability misclassification is possible, it is unlikely to have a major impact on the findings.

The third limitation to the findings stems form the use of routine District data, which have intrinsic low accuracy and reliability. However, the data on facilities' caseload and staffing were validated during ad-hoc supervision. The findings are consistent with those of other studies [24].

Another point is that the use of routinely collected data to map where women deliver in the District does not allow to take into account migration outside the District. HMIS in Tanzania is organized per District, and there are no records on whether women who deliver in facilities actually live in the District. It is possible that some women may have chosen to move beyond District boundaries or to private facilities for delivery, but these are likely to be wealthier women, thus the inequity observed may have in fact been greater.

A fifth point is that seasonal variations to access to the higher level facility are possible. Richer women may be disproportionately represented because they have the economic means to reach the hospital even when the roads are flooded. The major rain season in Iringa District is January to April, and hospital data was collected during both dry and rainy seasons, thus any effect should not be relevant.

Another limitation is that no variables measuring quality of maternal care was collected, such availability of equipment, supplies and knowledge. This has been examined in other studies [24], [30]. Insufficient staffing and volume of deliveries too low for staff to maintain skills are upstream challenges for first-line facilities, which make them unable to offer quality even if other requirements are fulfilled. There are no studies that have addressed the relation between caseload and quality of care in first-line facilities in limited resources countries. The definition of signal functions of basic and comprehensive emergency obstetric care [6] confirms that skills need practise to be maintained. To qualify as able to provide a signal function, facilities must have performed it at least once in the previous 3 months.

Lastly, no distinction was made among first-line facilities. Delivery services provided by dispensaries and health centres are very similar, and they share a role in fragmentation of deliveries, though health centres have greater numbers of skilled birth attendants (median 6.5, in dispensaries 1.5) and tend to attend greater numbers of deliveries.

Further studies, in particular on the relation between quality and caseload, will help to define the picture more accurately.

## Conclusion

In limited resources countries, when high coverage of facility deliveries is achieved, the poor remained disadvantaged. To tackle maternal mortality, a conflict between coverage and quality of delivery care in first-line health facilities should be addressed; the findings of the study suggest an adjustment of policy on where delivery care is available.

## Supporting Information

Dataset S1 **Study population dataset.**
(XLS)

Dataset S2 **Health facilities dataset.**
(XLS)
